# Pastoralism versus Agriculturalism—How Do Altered Land-Use Forms Affect the Spread of Invasive Plants in the Degraded Mutara Rangelands of North-Eastern Rwanda?

**DOI:** 10.3390/plants6020019

**Published:** 2017-05-12

**Authors:** Torsten Wronski, Jean Damascene Bariyanga, Ping Sun, Martin Plath, Ann Apio

**Affiliations:** 1 College of Animal Science and Technology, Northwest A&F University, Yangling 712100, China; mplath-zoology@foxmail.com; 2 Faculty of Science, School of Natural Sciences and Psychology, Liverpool John Moores University, Byrom Street, Liverpool L3 3AF, UK; 3 Department of Biology, College of Science and Technology, School of Science, University of Rwanda, P.O. Box 117, Huye, Rwanda; baridamass@gmail.com (J.D.B.); sunping9@icloud.com (P.S.); 4 Jomo Kenyatta University of Agriculture and Technology, Kigali Campus, P.O. Box 3373, Kigali, Rwanda; a-apio@gmx.de

**Keywords:** cattle grazing, goat browsing, living fences, *Dichrostachys*, *Cymbopogon*, grassland degradation

## Abstract

*Lantana camara* L. (Verbenaceae) originates from tropical Central and South America and has become invasive in about 50 countries. It causes problems when invading rangelands due to its toxicity to livestock and its tendency to form dense, monotonous thickets. Its invasiveness can partly be explained by the high tannin content largely protecting the species from being browsed, its tolerance to a wide range of environmental conditions, as well as its general preference for anthropogenically disturbed habitats. The dispersal of *L. camara* is facilitated by birds and other animals consuming its drupes (endozoochory), and so both wild and domestic ungulates could contribute to its spread. In our study, we investigated the distribution of *L. camara* in the Mutara rangelands of north-eastern Rwanda, an area that faced dramatic landscape changes in recent decades. We assessed 23 ecological factors and factors related to land-use and conservation-political history. Major effects on the local abundance of *L. camara* were found in that the relative canopy cover of *L. camara* was negatively correlated with the occurrence of other shrubs (suggesting competition for space and nutrients), while encounter rates of houses, ‘living fences’ (*Euphorbia tirucalli* L.) and cattle tracks were positively correlated with *L. camara* cover. Hence, the spread of non-native *L. camara* in the Mutara rangelands appears to be linked to landscape alterations arising from the transformation of rangelands supporting traditional pastoralist communities to other agricultural land-use forms.

## 1. Introduction

One of the major threats to global biodiversity is the spread of invasive species [1,2,3]. This applies usually to exotic (non-native) species that—once introduced and established in a new area—increase rapidly in local abundance and have negative impacts on the native flora or fauna [4]. However, not only exotic but also native species can become invasive when certain local environmental parameters change, e.g., due to human activities (altered land use [5,6,7]; logging [8]; environmental pollution [9]; artificially altered thermal regimes [10,11]), or as a consequence of economic trade [12,13]. In several cases, invasive species have a negative ecological impact on threatened or endangered native species [14,15], for instance, because invasive taxa cause competition for native taxa as seen between native and invasive *Impatiens* species in the Czech Republic [16]. Also, hybridization between native and introduced species can occur; e.g., invasive *Spartina alterniflora* Loisel. hybridizes with indigenous *Spartina foliosa* Trin. in the tidal marshes of San Francisco Bay, California [17], and invasive plants can alter the fire regime (e.g., cheatgrass, *Bromus tectorum* L. in western North America [18]), nutrient cycling (for review see [19]), or the hydrology of a given ecosystem (e.g., *Tamarix* sp. in the Lower Colorado drainage basin of Arizona [20]). Biological invasions can impact human livelihoods through environmental and economic costs arising from the uncontrolled spread of invasive species (e.g., in the United States of America an estimated 137 billion USD annually [15,21]).

Not all introduced (non-native) species become invasive though, and attempts to explain why a given species becomes invasive have focused on various ecological factors, including the release from natural enemies like herbivores or pathogens [22,23,24,25]. Also, high levels of phenotypic plasticity [26], and the potential for rapid evolutionary change [27] may contribute to a species’ invasiveness, as introduced species are unlikely to be ‘pre-adapted’ to several abiotic and biotic ecological parameters in their new range, such as the local climate, pollinator communities, herbivores, and so forth.

**Figure 1:**
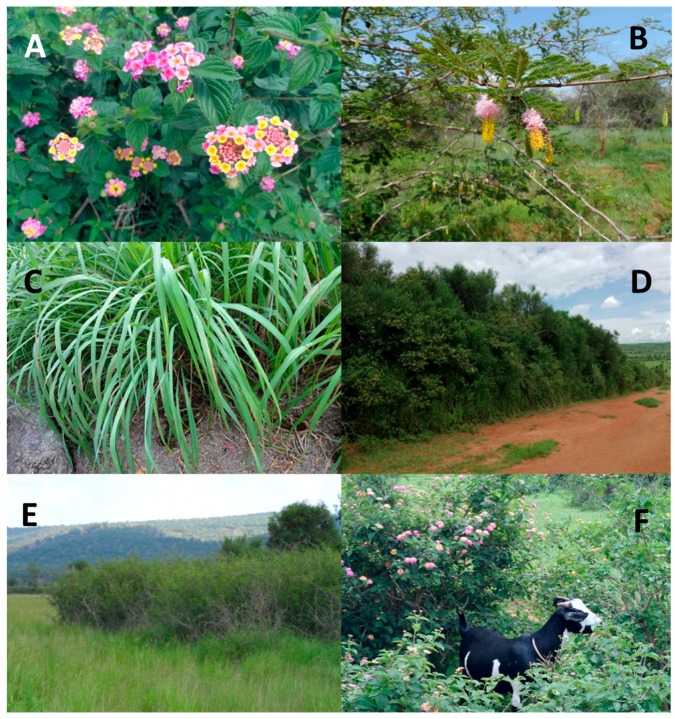
(**A**) Invasive *Lantana camara*, an exotic (non-native) shrub species from tropical South-central America in the Mutara rangelands; (**B**) an isolated stand of invasive *Dichrostachys cinerea* in Akagera NP; and (**C**) tufts of invasive *Cymbopogon nardus*, an unpalatable, aromatic Poaceae (© www.NatureLoveYou.sg); (**D**) living fence comprising of *Euphorbia tirucalli* and *L. camara*; (**E**) *Dichrostachys cinerea* invasion of a flood plain in Akagera NP; (**F**) domestic goat browsing on *L. camara* pods in the Mutara rangelands.

Our present study focuses on three invasive plants reported to negatively affect components of the agro-ecosystem located in the Mutara rangelands in north-eastern Rwanda [28,29]. We considered ecological factors that could be associated with the spread of non-native tickberry (*Lantana camara* L.; Verbenaceae; Figure 1A) originating from tropical Central and South America and the perennial grass species *Cymbopogon nardus* (L.) Rendle originating in tropical Asia (Poaceae; Figure 1C), as well as one native invasive species (i.e., indigenous species that became intrusive due to landscape or climate changes), namely *Dichrostachys cinerea* Wight et Arn. (Fabaceae; Figure 1B,E). *Lantana camara* has spread around the world, has become invasive in over 50 countries, and is currently becoming one of the most harmful invasive weeds in recorded history [30,31]. The invasiveness of *L. camara* can partly be explained by the high tannin content largely protecting the species from being browsed [32,33], its tolerance to a wide range of environmental conditions [34,35], as well as increased logging and other landscape modifications in conjunction with its general preference for anthropogenically disturbed habitats [36,37]. One of the most striking land-cover changes in rangelands during the last 150 years has been the proliferation of trees and shrubs at the expense of perennial grasses [38,39]. Shifts in the local abundance and overall density of woody plants represent fundamental habitat changes for herbivores—both wildlife (e.g., loss of mega-herbivores) and domestic livestock (e.g., introduction of cattle into previously ungrazed areas)—and hence lead to severe alterations in the ecosystems’ trophic structure. In the Mutara rangelands of north-eastern Rwanda, the native shrub *Dichrostachys cinerea* is increasing in abundance and density within its historic geographic range [40]. In semi-arid savannah habitats, such as the Akagera ecosysytem (to which the Mutara rangelands belong), drought, heavy grazing and fire suppression are suspected to lead to the encroachment of *D. cinerea* [41].

Two grass species are dominant in the Mutara rangelands: *Themeda triandra* Forssk. and *Hyparrhenia filipendula* (Hochst.) Stapf (both Poaceae). In places, these two are replaced by invasive *C. nardus*, which is strongly aromatic, and both domestic and wild ungulates avoid it [42]. *Cymbopogon nardus* is an invasive grass species in the rangelands of eastern and southern Africa, affecting the growth of desirable, palatable grass species and impeding movement of livestock. In India, the occurrence of *Cymbopogon* sp. is typically linked to frequent burning [43], but in Africa (including the Mutara) it appears to grow better in the prolonged absence of fire [40,44,45,46]. However, its occurrence in the Mutara seems to be linked to overgrazing or to soil disturbance caused by previous agricultural activities [40].

The Mutara rangelands harbor a large traditional pastoralist community. Today’s rangelands originate from the degazetted parts of Akagera National Park, the Mutara Game Reserve and three communities from adjoining sectors that were never protected. Since 1997, a resettlement program has resulted in a major influx of returning refugees and their livestock [47,48,49]. Today, pastoral and agro-pastoral farming systems dominate in the Mutara, accounting for one-third of Rwanda’s human population and for 85% of the country’s cattle. To manifest land-ownership, the remaining rangeland (app. 13% of land area; results from this study) is increasingly divided by living fences comprising of *Euphorbia tirucalli* and lately of unintentionally sowed *L. camara* (Figure 1D). In recent years, cattle is increasingly replaced by domestic goats in an attempt to diversify local livelihoods [50]. Beside several avian species consuming the berry-like drupes, rodents and primates are the only mammals that regularly consume *L. camara* and may thus contribute to the dispersal of this invasive shrub [51,52,53]. However, there are also reports of seeds being dispersed by sheep and goats (Figure 1F) [54].

In our study we asked what factors drive the spread, especially of non-native *L. camara*, but also native invasive *D. cinerea* and non-native *C. nardus* in the Mutara rangelands, which may provide vital information regarding the question of how to impede their further spread. We first asked whether wildlife (i.e., birds, primates or ungulates) could be a major factor facilitating their dispersal [51,52,53]. In this case, the distribution of invasive plants should correspond with high wildlife densities or bird species richness. We also tested whether domestic livestock contributes to the distribution of invasive plants ([54], but see [32,33]), in which case percentage cover of invasive plants should positively correlate with local livestock densities. Third, we predicted human landscape alterations (i.e., construction of fences, roads, houses and gardens) to be partly responsible for the spread of invasive plants [36]. In that case, invasive plant cover should increase with increasing abundance of anthropogenic structures. In our study, we therefore assessed various ecological factors across 44 study quadrants as well as factors related to land-use and conservation-political history in the Mutara rangelands (independent variables). We condensed them into seven principal components (PCs) and tested for their impact on the distribution and local abundance of the three invasive plant species (dependent variables).

## 2. Results

In the case of *Lantana camara* and *Cymbopogon nardus*, we found significant effects of environmental variables, condensed into six PCs (Table 1), while percentage cover of *Dichrostachys cinerea* was not affected by any PC (Table 2). ANCOVAs (General Linear Models, GLM) revealed that percentage cover of *L. camara* and *C. nardus* were significantly affected by PC2, which received axis loadings (>|0.50|) from tree cutting, charcoal burning, shrub canopy cover and grass frequency (Table 1). Percentage cover of *L. camara* was also influenced by PC3 and the interaction term ‘PC3 × PC6’ (Table 2). PC3 received high axis loadings from human and house densities, living fences and watering troughs. The interaction term ‘PC3 × PC6’ received additional axis loadings from cattle tracks and erosion (Table 1). Visualizing the interaction effect yielded somewhat different slopes of the two regressions when splitting the data by the median of PC3; in both cases, the same pattern became apparent, i.e., percentage *L. camara* cover decreased with increasing PC6 (Supplementary Materials, Figure S1).

**Figure 2:**
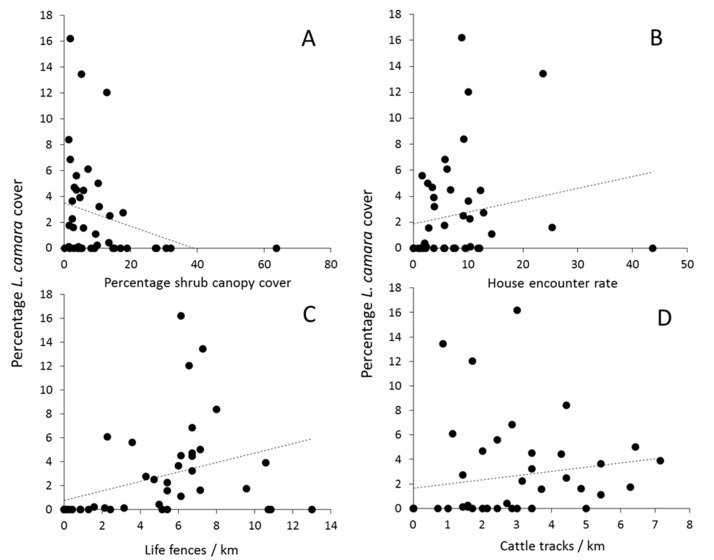
Post-hoc Spearman rank correlations for significant effects between percentage *Lantana camara* cover and (**A**) percentage shrub canopy cover; (**B**) house encounter rate; (**C**) living fences and (**D**) cattle tracks. Linear fits were included to show the tendency (positive or negative) of relationships.

We used post-hoc analyses based on Spearman’s *ρ* to test the robustness of the effects detected in our GLMs. We found significant effects for the correlations between relative *L. camara* canopy cover and percentage shrub canopy cover, house encounter rates, numbers of living fences and numbers of cattle tracks (Table 3). The relative canopy cover of *L. camara* was negatively correlated with the occurrence of other shrubs (Figure 2A), while house encounter rate, and numbers of living fences and cattle tracks were positively correlated with *L. camara* cover (Figure 2B–D). The correlation between relative *L. camara* canopy cover and incidences of soil erosion was marginally non-significant (*r* = 0.276, *p* = 0.069; Table 3). In the case of *C. nardus*, however, none of the post-hoc correlation analyses were significant (Table 3).

## 3. Discussion

Because of its invasiveness, potential for rapid spread once arrived in a new area, and economic and environmental impacts arising from its invasiveness, *L. camara* is regarded as one of the worst invasive weeds in tropical grasslands [31,55]. Due to its ability to form dense, impenetrable thickets that take over native savannah bush- and grasslands it competes for resources, reduces the productivity of pastures and thus the condition of livestock. Our study revealed that the occurrence of *L. camara* is indeed negatively correlated with the occurrence of other shrubs in the Mutara rangelands, and that the abundance of houses, living fences and cattle tracks is positively correlated with *L. camara* cover. The negative correlation between *L. camara* cover and that of other shrubs, such as *Maerua angolensis* DC., *M. triphylla* A. Rich., *Capparis tomentosa* Lam. (Capparaceae), *Pavetta gardeniifolia* Hochst. ex A. Rich. (Rubiaceae), *Grewia similis* L., *G. mollis* Juss. (Malvaceae), or *Carissa edulis* (Forssk.) Vahl. (Apocynaceae), confirms competition for space and resources between *L. camara* and those species in the Mutara rangelands. *Lantana camara* is known to produce toxic chemicals that prevent competing plant species from flourishing [55]. Also, the extraordinarily high seed production of *L. camara* (app. 12,000 seeds from each plant per year [37,55] might explain the negative correlation between *L. camara* and other (native) woody shrubs and bushes in the Mutara rangelands. A possible reason for the spread of *L. camara* in the Mutara rangelands may be the species’ ability to alter the soil nitrogen content [56], thereby decreasing the competitiveness of other shrubs, especially members of the (nitrogen-fixing) family Fabaceae. This will need to be investigated in future studies that adequately capture additional ecological parameters like soil nitrogen contents.

As predicted [36], *L. camara* was more abundant where the number of houses, cattle trails and living fences was high, i.e., in areas that are increasingly dissected by living fences (to prevent cattle from entering farms or other ranches). Living fences are usually encountered close to settlements and villages, where landownership is dense and formerly continuous rangelands are nowadays highly fragmented. It seems, therefore, that intensified land-use following the return of war refugees after 1995 and a shift from traditional pastoralism to small-scale subsistence agriculture promotes the spread of *L. camara* in the Mutara rangelands. By contrast, the two native invasive plant species considered in this study, i.e., *D. cinerea* and *C. nardus* were not affected (i.e., not more prevalent in association with anthropogenic disturbance) to the same extent by anthropogenic habitat alteration as *L. camara*. Still, in our analyses of variance using principal components as predictor variables (but not in post-hoc correlation analyses), an effect of PC2 on the local abundance of *C. nardus* was found, suggesting that it shows some trend towards a similar pattern as observed for *L. camara*: a negative correlation between relative *C. nardus* cover and native shrub species abundance and a positive correlation between *C. nardus* cover and the abundance of houses, living fences and cattle tracks. As predicted by [40], the occurrence of *C. nardus* in the Mutara rangelands seems to be linked to overgrazing (cattle tracks) or to soil disturbance caused by previous (and current) agricultural activities (living fences, house abundance). However, future studies using more sample quadrants and/or a different scale at which ecological parameters are assessed (i.e., smaller sampling quadrants) may be needed to identify factors predicting the spread of *C. nardus*, as our study design may not have been appropriate to adequately capture the (weak) effects of different ecological factors considered herein. Absence of a statistically significant effect in our present study, of course, does not prove that the respective set of ecological factors has no effect; rather our study may suffer from insufficient sample sizes in combination with moderate to weak effect strengths. Moreover, this study was initiated from a wildlife-oriented perspective, and we secondarily shifted our focus towards the spread of invasive plants in the area, but future studies may be needed to consider additional factors of relevance from a botanical perspective like the nitrogen-fixing ability of *D. cinerea* or the accumulation of nitrogen in the soil, as reported for *L. camara* [56].

A number of reasons have been put forward to explain why *L. camara* has been particularly successful as an invasive species [37]. The high tannin content protecting the species from being browsed [32,33], its ability to disperse using birds as vectors [51] (as well as endozoochory by other animals consuming its drupes [54]), and its general tolerance to a wide range of ecological factors in anthropogenically disturbed habitats appear to be prime reasons for the species’ high invasion potential [34,35,36,57,58,59]. Moreover, it was reported that the digging activity of ungulates, such as pigs, enhances the dispersal of *L. camara* in Australia by causing the death of trees and subsequent increased light penetration, which favored the spread of *Lantana* [57]. In the Mutara rangelands, land-use changes and habitat modifications are mainly caused by increasing subsistence agriculture and high cattle densities (up to 1200 cattle km^−1^, this study). In our analysis, these forms of anthropogenic disturbance were reflected by significant effects of encounter frequencies of houses, living fences and cattle tracks. However, it remains unclear why cattle densities as such did not have a direct significant effect on *L. camara* cover, but only numbers of cattle tracks. Possibly, the species is more resistant to soil disturbance resulting from permanent cattle movement than many native shrub species.

Pastoralism is an important economic and cultural way of life for 100–200 million people worldwide [60,61]. Extensive pastoral systems cover about 25% of the earth’s terrestrial surface, while in sub-Saharan Africa, about 16% of the human population relies entirely on pastoralism [62,63]. Pastoralism is characterized by low human population densities, high mobility and high dependency on local ecological knowledge [64,65]. Human activities in pastoralist communities contribute to the production and stability of the ecosystem, e.g., livestock grazing influences soil fertility [66], distribution and diversity of plants, maintains natural vegetation [67], captures carbon [68], reduces erosion, maintains soils, maintains the water holding capacity of the soil and provides habitat for wildlife [58,69,70]. Due to close links between pastoral communities, the ecosystems in which they live, and the animals they breed, pastoralism plays a significant role in the conservation and sustainable use of biodiversity [70,71,72]. Only in recent decades (i.e., since 1997) the Mutara rangelands faced dramatic land-use changes [47], mainly by dissecting the natural habitat with living fences to separate cattle from agricultural gardens and to secure landownership. Maintaining a traditional pastoralist system would not only benefit wildlife and biodiversity [72], but, as our study suggests, could also help reduce the spread of invasive *L. camara*. To prevent further habitat degradation in this part of the Akagera savannah ecosystem it would be advisable to prevent *L. camara* from thriving in living fences and to discourage pastoralist communities to tolerate it on their ranches. Contrary to our original prediction, the data at hand suggest that goat browsing does not play a major role in the spread of *L. camara*. Obviously, this finding does not imply that goats would prevent the spread of *L. camara*; however, it provides another argument for local pastoralists to increasingly replace cattle by goats to avoid overstocking and erosion, but also to diversify local livelihoods. Altogether then, our findings may help to confine the spread of *L. camara* while at the same time promoting the persistence of wildlife and livelihood diversification through goat breeding. Intensified land-use appears to be one of the main factors promoting the spread of invasive *L. camara*, and land over-exploitation is not only due to agriculatural practices, but partly linked to the ever intensifying use of land for cattle grazing. Goat breeding has been suggested as a sustainable alternative for the local human community, as it prevents bush encroachment (goats are predominantly browsers) and reduces zoonotic disease transmission between wildlife and livestock species and between livestock and humans [73].

## 4. Material and Methods

### 4.1. Study Area

**Figure 3:**
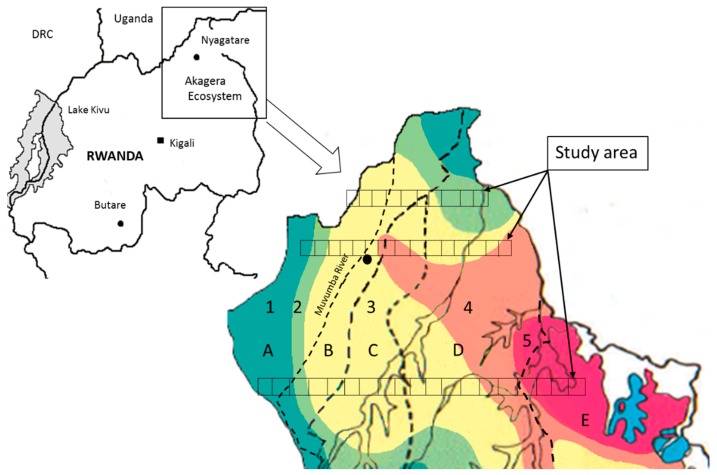
Our study area (forty-four 2.5 × 2.5 km squares) extending along three transect belts across the Mutara rangelands in the Akagera ecosystem in Rwanda. Conservation-political history and eco-climate (modified from [40] and separated by dashed lines) are: (A, west) Mutara rangelands, never protected, (B, central west) Mutara Game Reserve, degazetted between 1971 and 1990, (C, central) Mutara Game Reserve, degazetted in 1997, (D, central east) former Akagera National Park, degazetted in 1997, (E, east) modern Akagera National Park; (1, dark green) below Byumba Escarpment (900–1000 mm annual precipitation), (2, light green) higher Mutara (800–900 mm), (3, yellow) lower Mutara (700–800 mm), (4, orange) drier Mutara (600–700 mm), (5, pink) drier Kagera (500–600 mm).

The Mutara rangelands are situated in the Nyagatare District of north-eastern Rwanda (Figure 3) and—together with the Kagera District in Tanzania and the Ankole grasslands in south-western Uganda—are part of the Akagera ecosystem. The Mutara rangelands comprise vast open grasslands and savannah woodlands that are predominantly used for grazing cattle. The natural woody vegetation is dominated by compound-leafed trees such as *Senegalia senegal* (L.) Willd. and *S. polycantha* (Willd.) Seigler & Ebinger (Fabaceae), and broad-leafed species like *Combretum* spp. (Combretaceae), while grasslands are mostly composed of gramineous species such as *Bracharia* spp., *Hyparrhenia filipendula* (Hochst.) Stapf, *Themeda triandra* Forssk. and *Sporobolus pyramidalis* Beauv. (Poaceae) [40,74]. However, during the last two decades rangelands were increasingly transformed into subsistence agriculture to grow banana, maize and legumes.

In July 1934, large parts of the Mutara rangelands were protected as part of the newly gazetted Akagera National Park or the Mutara Game Reserve [40]. Between 1973 and 1990 the western parts of the Mutara Game Reserve (east of the Muvumba River; Figure 3) were opened for development projects, livestock breeding and the army [40]. Following the civil war (1991–1995), the remaining Mutara Game Reserve and the western half of Akagera National Park were also degazetted, reducing the protected area from an initial surface area of 2800 km^2^ to about 1120 km^2^ [75,76,77]. Other areas that were also included in our study, such as the area west of the Muvumba River, i.e., Rwempasha, Tabagwe and Rukomo Sectors, were never protected.

Sampling was carried out along three transect belts across the Mutara rangelands, covering five different zones of conservation-political history from the international border with Tanzania (or the wetlands inside the modern Akagera National Park) in the East to the international border with Uganda (or the Byumba Escarpment) in the West (Figure 3). Transect belts were established as described in [78], while omitting the two westernmost quadrants from the central and the southern transect belt, resulting in a total of 44 quadrants each measuring 2.5 × 2.5 km.

### 4.2. Assessment of Invasive Plant Species (Dependent Variables)

We assessed the relative ground cover [%] of non-native *Lantana camara* L. (Verbenaceae), native invasive *Dichrostachys cinerea* Wight et Arn. (Fabaceae) and non-native *Cymbopogon nardus* (L.) Rendle (Poaceae) using the line intercept method [79,80]. To eliminate potential seasonal variation, percentage cover was measured 40 times (i.e., 20 times in the wet season, 20 times in the dry season at different locations) in each quadrant. Percent cover was established by recording the length of intercept for each plant type along a haphazardly stretched tape (50 m) while measuring the distance between starting and ending points of the respective vegetation type. Intercept lengths for a given species were summed and divided by the total tape length. We averaged data from all 40 measurements per quadrant. Using the same method, we simultaneously established the percentage cover for trees and shrubs other than the three invasive species to use them as ecological predictor variables (see below).

### 4.3. Ecological Predictor Variables (Independent Variables)

#### 4.3.1. Conservation-Political History and Eco-Climatic Data

Assignment of quadrants to different conservation historical and eco-climatic zones followed [40] (Figure 3). Ten quadrants were situated in those parts of the Mutara rangelands that were never protected, six in parts of the Mutara Game Reserve that were degazetted between 1973 and 1990, eight in parts of the Mutara Game Reserve that were degazetted in 1997, 16 in the former Akagera NP (degazetted in 1997) and four in the modern Akagera NP. Eco-climatic zonation was based on rainfall data obtained before 1990. Three quadrants were located in the wettest zone (900–1000 mm annual precipitation) near the Bujumba Escarpment in the West, six in the higher Mutara rangelands (800–900 mm), 26 in the lower Mutara rangelands (700–800 mm), five in the drier Mutara rangelands (600–700 mm) and four in the dry forest (500–600 mm) inside the modern Akagera National Park.

#### 4.3.2. Livestock Density

Overstocking with domestic livestock is often held accountable for increasing erosion, soil compaction and loss of plant cover [81]. Disturbed soil and changes in the plant community can facilitate the spread of invasive plant species [36]. Occasionally, endozoochory, i.e., the dispersal of seeds after passage inside an animal’s digestive tract, could also play a role in the dispersal of invasive plants (e.g., *Prosopis juliflora* (Sw.) DC., Fabaceae [82]). We established three walking transects in each quadrant (two 1.5 km and one 0.5 km long) to count the number of local Ankole cattle as well as goat and sheep. Transect counts were conducted once in the dry season and once in the wet season, and averaged data were used for subsequent analyses. For distance sampling analysis, the perpendicular distance was determined for each livestock encounter, i.e., the direct distance between the transect line and the animal or group of animals, using a *Bushnell Yardage ProX 500* range finder. To estimate livestock densities, we employed the software DISTANCE vs. 6.0 [83,84]. Making full use of the power of Akaike Information Criteria (AIC) and information theory, we analyzed cattle, goat and sheep together; whereby, due to low sampling sizes, goats and sheep were pooled into one category. We first explored the full data set to determine the best-fitting model, i.e., the detection function with the lowest AIC (Table S1, Supplementary Materials). Raw distances were binned into three or four intervals (avoiding cut points that coincide with multiples of 10) and truncated at 25–350 m to eliminate outliers from the model. All other binning intervals or truncations that resulted in smaller, i.e., significant χ^2^-values were omitted. Subsequently, we split the overall data set into four groups (cattle in the dry season, cattle in the wet season, sheep + goat in the dry season, sheep + goat in the wet season), rerunning the analyses with the same settings as established above. Details on key functions, series expansion, AIC, effective strip width (ESW), number of detections, mean cluster size, density of individuals, standard error of density, 95% confidence interval, percent coefficient of variation, and the density of clusters are provided in Table S1 (see Supplementary Materials).

#### 4.3.3. Wildlife Encounters Frequency and Bird Richness

Although their high tannin content is suspected to largely protect *L. camara* and *C. nardus* from being eaten [32,33,42,44,46], it can be assumed than at least some wildlife species (browsing ungulates and primates) consume parts of these plants and thus contribute to their dispersal. Wildlife densities were estimated using the local ecological knowledge of the resident pastoralist population following previously established methods [78]. The interview survey involved a total sample of 526 independent participants. Only persons were selected for interviews who either worked on their own land (gardeners), who claimed to own the adjacent cattle ranch (cattle owners), who frequently graze their own (or another person’s) cattle in the area (herdsmen), or who stated that they continuously collect fire wood on that land. We interviewed respondents at the spot (in their garden, on their ranch or occasionally within their homes), using a semi-structured questionnaire (Supplementary Materials, Questionnaire 1). Each interview took around 30 min to be completed. We targeted only permanently resident adults (>18 years old) as respondents, and a balanced gender ratio was aspired but not entirely achieved [211 (40.1%) male and 315 (59.9%) female participants]. We took GPS coordinates of the interview site using a Garmin GPS III to ensure that places were situated within the respective quadrant. Interviews were carried out by one interviewer conversant in the local language (Kinyarwanda).

We presented silhouette images of 21 ungulate species and three primate species potentially occurring in the study area to each respondent. Eight ungulates (common duiker, *Sylvicapra grimmia*; bushbuck, *Tragelaphus scriptus*; oribi, *Ourebia ourebi*, sitatunga, *Tragelaphus spekeii*; bohor reedbuck, *Redunca redunca*; impala, *Aepyceros melampus*) and two primates (olive baboon, *Papio anubis*; vervet monkey, *Cercopithecus aethiops*) were identified to still occur in the area outside modern Akagera NP. The interviewees were first asked whether they ever encountered each species on their land. Subsequently, we asked for the abundance of each species, i.e., the frequency each species was encountered during the last year (on how many days per year the interviewee encountered that species). Encounter frequencies of the eight ungulates (ungulate encounter frequency) and the two primates (primate encounter frequency) were then summed for each quadrant and divided by the number of respondents in that quadrant. For the quadrants inside the modern Akagera NP, ungulate and primate frequencies were assigned a maximum ceiling value of 365 days, i.e., corresponding to an interview answer of encountering those species every day.

The berries of *L. camara* are dispersed by several bird species [85] enabling *L. camara* to expand its range rapidly and to occupy a broad range of environments. Moreover, *Dichrostachys cinerea* seeds are consumed by numerous bird species (at least as secondary consumers from ungulate dung [86]), while *Cymbopogon* seeds are usually not consumed by birds [87,88]. Since detailed information on what bird species consume certain plant parts in the Akagera ecosystem is lacking, we decided to determine overall bird species richness (rather than abundance estimates for certain consumers) in each quadrant, and transect belts were fitted to the presence/absence grid for each bird species occurring in Rwanda provided by [77]. The number of bird species reported by [77] for each grid was transferred to the corresponding quadrants of our transect belts and applied to all relevant quadrants. Usually, three quadrants of this study coincide with one grid cell in [77], implying same species counts for those quadrants.

#### 4.3.4. Degree of Grassland Fragmentation

To assess the extent to which rangeland was transferred into areas of agricultural use, we measured the ratio of cattle ranches (grassland savannah) to gardens (subsistence agriculture). Waypoints for each change from a garden to a ranch and vice versa were taken whilst walking transects established to count domestic livestock species (see above). Later, we plotted location fixes using BASE CAMP software (Garmin) to determine the distance between measuring points. We expressed data as proportions of garden or ranch cover (km garden or ranch/total transect length). Ranches (grassland) and gardens are the two major land use forms encountered in the study area. For Akagera National Park, the degree of grassland fragmentation was set as 100% grassland (i.e., 3.5 km ‘ranch’), as parts of Akagera National Park were used for ranching prior to the designation as a National Park in 1934.

#### 4.3.5. Human Disturbance

We established two variables for each quadrant to estimate the degree of direct anthropogenic impact, i.e., the encounter rates of houses and humans. We counted the number of houses (within a strip of 150 m to both sites of the route) and the number of people encountered along a representative route (1.5 km) within each quadrant (any public track or road). The human encounter rate is indicative of the intensity of ‘traffic’, while the house encounter rate denotes the number of people effectively settling in that area. Furthermore, while walking transects to count livestock, we also counted the number of living fences (*Euphorbia tirucalli*) planted by ranch and garden owners to protect crops and pasture from unauthorized grazing, the number of cases of soil erosion on the transect, the number of cattle tracks crossing each transect line, the number of watering troughs to provide cattle with water, as well as numbers of incidences of tree cutting and charcoal burning, and divided all count data by the distance travelled (i.e., 3.5 km) to obtain the encounter rate.

#### 4.3.6. Water-Holding Capacity and Soil Porosity

We assessed two soil parameters that provide information on the degree of soil compaction. Soil compaction can be related to overstocking with domestic livestock but also to increasing urbanization [89]. Both types of environmental changes are described to have an impact on the dispersal and abundance of invasive plant species [36,81]. We collected soil samples of 261.25 cm^3^ volume using a cylindrical soil core (23.75 cm^2^ ground area × 11 cm height) at ten randomly selected locations per quadrant and transferred samples to the laboratory to obtain the wet and dry weight. Water-holding capacity and soil porosity (aeration porosity) were calculated as follows: wet soil weight minus dry soil weight gives the mass of contained water (gravimetric water content). Assuming that the density of water is 1 g cm^−3^, we thus obtained the volume of water. Dividing the volume of water by the surface area of the container gives the depth of water. Dry soil weight divided by the particle density of 2.65 g cm^−3^ gives the soil volume. By dividing the soil volume by the surface area of the container we obtained the depth of soil. Subtracting the depth of water and the depth of soil from the height of the container provides the depth of air. The water-holding potential was then calculated by dividing the mass of water and air by the mass of soil × 100. To obtain soil porosity, we divided the depth of air by the height of the container × 100.

#### 4.3.7. Above-Ground Monocotyledonous Biomass

As an indirect measure of overstocking with cattle and, therefore, degradation of rangeland due to overgrazing, the above-ground monocotyledonous plant biomass was determined as described in [90]. All above-ground grass matter was cut in twenty 50 × 50 cm plots (10 in the dry season, 10 in the wet season) in each quadrant. Samples were processed in the laboratory by measuring the wet weight and then drying the grass sample to obtain the dry weight. To obtain ash-free dry weight (AFDW; grass biomass excluding water and minerals), the dried material was oxidized (ashed) in a muffle furnace and re-weighed.

#### 4.3.8. Grass and Herb Frequency

The frequency of other gramineous species is negatively correlated with the occurrence of *C. nardus* in the rangelands of south-western Uganda [44,46]. Moreover, the decline (or increase) of herb abundance and diversity in a savannah ecosystem was reported to be a good indicator for the degradation of rangelands [91,92,93,94]. Grass and herb frequencies were measured 20 times in each quadrant (10 times in the dry season, 10 times in the wet season) following methods described in [80]. Sampling locations (only situated in grassland) were roughly identified in Google Earth, while a random procedure was applied on site to determine the final sampling spot. A 0.9 × 0.9 m sampling rectangle was laid arbitrarily on the ground and the presence of grass or herbs in each sampling grid recorded to establish the fraction of grids containing grass or herbs.

### 4.4. Data Analysis

In all cases, measurements were averaged across samplings and seasons to obtain one value for each quadrant. Prior to statistical analyses we arcsine (square root)-transformed all relative data (*L. camara*, *D. cinerea*, *C. nardus*, as well as tree and shrub canopy cover, cattle and sheep/goat densities, ungulate and primate encounter frequencies, bird species richness, house and people densities, ranch/garden ratio, canopy cover of shrubs and trees, as well as grass and herb frequencies). Subsequently, we applied *z*-transformation to the entire data set to standardize data dimensionality. Inspection of model residuals did not indicate violations of model assumptions, i.e., normal error distribution and homoscedasticity. Multiple correlation analysis between our three dependent variables (i.e., local abundances of the three invasive plant species per quadrant) and all 23 independent variables would have required Bonferroni-correction of significance thresholds (α-levels) to avoid type I errors as: α’ = α/23 = 0.05/23 = 0.0022. To avoid restrictions arising from this approach, we decided to condense all explanatory variables through a factor reduction (principal components analysis, PCA, based on a correlation matrix) using the varimax rotation option. The six resulting principle components with an eigenvalue >1.0, explaining 74.2% of the total variance (Table 1) were then used as explanatory variables in our statistical analyses.

We tested what factors predict the local abundance of the invasive plant species by including the six PCs as covariates in three independent General Linear Models (GLMs, one for each species), in which percent cover of each species was specified as dependent variable. We initially included all two-way interactions of covariates in the GLMs. Interaction terms were excluded if *p* > 0.1 (all excluded terms: *F* < 2.61, *p* > 0.12). We also initially included ‘transect ID’ as a random factor in all analyses, but removed it from the final models as the effects were not significant (*F* < 2.87, *p* > 0.093).

To avoid over-interpretation of our results, we tested the robustness of statistically significant effects in our GLMs via post-hoc non-parametric Spearman rank correlations with those factors contained in the respective PCs that received high axis loadings (>|0.50|; Table 1). Our main conclusions are based on significant results in our post-hoc analyses.

## Figures and Tables

**Table 1:** Results of principal component analysis of the 23 explanatory variables. Variables were measured in 44 quadrants along three transect belts across the Mutara rangelands. PC loadings > 0.5 are shown in bold font type.

Variable	PC1	PC2	PC3	PC4	PC5	PC6
Eigenvalue	5.43	3.75	2.74	2.11	1.68	1.35
% variance explained	23.62	16.29	11.90	9.18	7.31	5.89
Eco-climate	**0.825**	−0.202	−0.023	0.244	−0.027	0.202
Conservation-political history	**0.648**	0.076	−0.351	−0.015	−0.416	0.029
Cattle density	−0.053	0.198	0.021	**0.760**	0.137	0.043
Goat density	−0.066	0.025	−0.100	0.333	**0.744**	0.386
Ungulate encounter frequency	**0.906**	−0.005	−0.134	−0.113	−0.012	−0.172
Primate encounter frequency	**0.883**	0.033	−0.076	−0.214	0.066	−0.140
Bird species richness	**0.587**	0.002	−0.140	0.236	−0.349	0.286
House encounter rate	−0.247	−0.080	**0.855**	−0.082	−0.137	0.068
Human encounter rate	−0.287	−0.034	**0.854**	−0.056	−0.076	−0.151
Ranch/garden ratio	**0.829**	−0.103	−0.197	−0.095	0.069	−0.057
Living fences	−0.212	−0.288	**0.569**	0.434	0.039	0.471
Cattle tracks	−0.201	−0.012	0.217	0.456	−0.015	**0.667**
Tree cutting	0.125	**0.753**	−0.158	0.352	−0.274	0.245
Charcoal burning	0.136	**0.776**	0.012	0.258	−0.100	0.074
Watering troughs	0.024	0.093	**0.689**	0.356	0.300	0.297
Erosion	−0.101	0.072	0.018	−0.053	−0.026	**0.799**
Tree canopy cover	**0.606**	0.397	−0.117	0.100	−0.091	0.310
Shrub canopy cover	−0.219	**0.727**	−0.132	−0.123	0.062	−0.171
Monocotyledonous biomass	−0.231	0.391	−0.036	0.004	**0.539**	−0.309
Grass frequency	0.096	**−0.642**	−0.212	0.494	−0.140	−0.077
Herb frequency	−0.018	0.015	−0.066	**−0.751**	0.252	−0.101
Soil water holding potential	−0.019	−0.068	0.061	−0.093	**0.668**	−0.460
Soil porosity	0.056	−0.231	−0.050	−0.323	**0.728**	0.068

**Table 2:** Results of GLMs using the seven eco-climatic, conservation history-related and ecological principal components (PCs) as covariates. Interaction terms were excluded if *p* > 0.1.

Factor	df	Mean Square	*F*	*p*
*Lantana camara*				
PC1	1	1.454	2.291	0.139
PC2	**1**	**3.150**	**4.964**	**0.032**
PC3	**1**	**3.899**	**6.145**	**0.018**
PC4	1	1.491	2.350	0.134
PC5	1	0.003	0.005	0.943
PC6	1	0.853	1.345	0.254
PC3 × PC6	**1**	**9.334**	**14.712**	**0.0001**
Error	36	0.634		
*Dichrostachys cinerea*				
PC1	1	0.799	0.760	0.389
PC2	1	2.018	1.919	0.174
PC3	1	0.079	0.075	0.786
PC4	1	0.569	0.541	0.467
PC5	1	1.235	1.175	0.285
PC6	1	0.399	0.380	0.542
Error	37	1.051		
*Cymbopogon nardus*				
PC1	1	2.223	2.284	0.139
PC2	**1**	**4.769**	**4.901**	**0.033**
PC3	1	0.281	0.289	0.594
PC4	1	0.105	0.108	0.745
PC5	1	0.326	0.335	0.566
PC6	1	0.289	0.297	0.589
Error	37	0.973		

**Table 3:** Post-hoc Spearman rank correlations between percentage cover of *Lantana camara* (as well as *Cymbopogon nardus*) and factors with high axis loadings (see Table 1) contained in PC2, PC3 and PC6.

***Lantana camara***				
PC2	Tree Cutting	Charcoal Burning	Shrub Canopy Cover	Grass Frequency
*r*	+0.147	−0.071	**−0.348**	+0.014
*p*	0.341	0.648	**0.021**	0.926
PC3	House encounter rate	People encounter rate	Living fences	Watering troughs
*r*	**+0.333**	+0.211	**+0.558**	+0.227
*p*	**0.027**	0.167	**<0.0001**	0.137
PC6	Cattle tracks	Erosion		
*r*	**+0.398**	+0.276		
*p*	**0.008**	0.069		
***Cymbopogon nardus***				
PC2	Tree cutting	Charcoal burning	Shrub canopy cover	Grass frequency
*r*	+0.203	+0.097	+0.157	−0.172
*P*	0.184	0.532	0.307	0.264

## Supplementary Materials

The following are available online at www.mdpi.com/2223-7747/6/2/19/s1, Figure S1: Visualization of PC3 × PC5 interaction effect on relative canopy cover of *L. camara*, Table S1: Details of DISTANCE analysis of seasonal cattle and sheep/goat densities, Questionnaire 1: Semi-structured questionnaire.
