# Supplementary material for: Pastoralism versus Agriculturalism—How Do Altered Land-Use Forms Affect the Spread of Invasive Plants in the Degraded Mutara Rangelands of North-Eastern Rwanda?
Source: Plants (Basel). 2017 May 12;6(2):19. doi: 10.3390/plants6020019 (PMC5489791; doi:10.3390/plants6020019)
Supplement: Supplementary file 1 [file plants-06-00019-s001.pdf]

## Online Supplementary Material

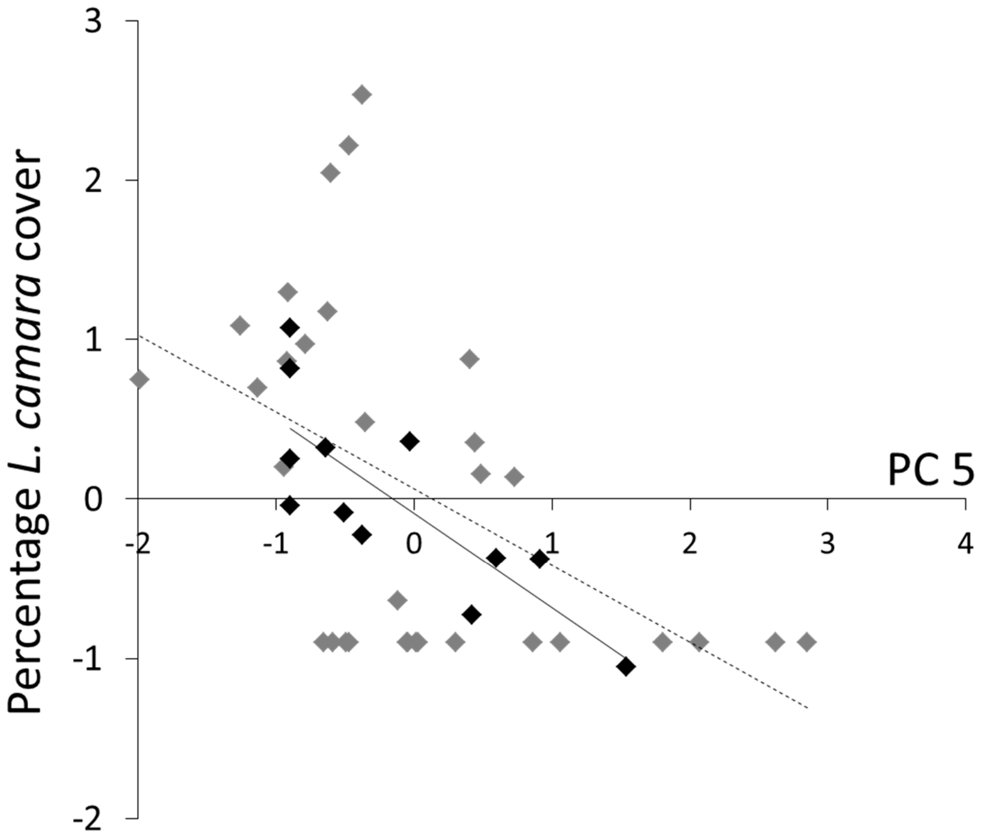


**Figure S1.** Visualization of the interaction effect of PC3 × PC5 on relative canopy cover of *L. camara*. Decreasing cover of *L. camara* (arcsine sqrt and afterwards *z*-transformed values) along PC5 becomes evident for both cohorts of data when splitting the data by the median of PC3 (dashed line: PC3 > median; solid line: PC3 < median).

**Table S1.** Details of DISTANCE analysis of seasonal cattle and sheep/goat densities in 44 sampled quadrants. Given are best fitting models including key functions, series expansion, Akaike Information Criteria (AIC), $\chi^2$, Effective Strip Width (ESW), as well as numbers of detections (*N*), mean cluster size ($\hat{Y}$), density of individuals (D), standard error of density (D(SE)), percent coefficient of variation (V%), 95% confidence interval (CI 95%), and the density of clusters ($D_g$).

| Quad. | model | AIC | $\chi^2$ | ESW | species | season | *N* | $\hat{Y}$ | D | D (SE) | V% | CI 95% | $D_g$ |
|---|---|---|---|---|---|---|---|---|---|---|---|---|---|
| **C3d** | Halfnorm.-cosine | 33 | 0.3 | 53 | cattle | dry | 7 | 12.1 | 171.9 | 101.8 | 59.2 | 48-616 | 9.3 |
| | | | | | | wet | 9 | 18.0 | 316.9 | 218.8 | 69.0 | 78-1282 | 12.0 |
| | | | | | sheep/goat | dry | 5 | 8.8 | 717.0 | 538.7 | 751 | 24-591 | 6.7 |
| | | | | | | wet | 4 | 6.2 | 99.4 | 106.0 | 106.7 | 11-915 | 5.3 |
| **D3c** | Halfnorm.-cosine | 31.2 | 0.52 | 68 | cattle | dry | 5 | 14.4 | 20.3 | 13.7 | 67.6 | 5-83 | 7.3 |
| | | | | | | wet | 7 | 17.4 | 71.1 | 50.9 | 71.6 | 18-283 | 3.1 |
| | | | | | sheep/goat | dry | 3 | 15.3 | 379.7 | 1074.0 | 282.9 | 0-98 | 3.1 |
| | | | | | | wet | 1 | 0.0 | 1.1 | 1.0 | 98.3 | 0-8 | 1.1 |
| **D3d** | Halfnorm.-cosine | 40.7 | 0.34 | 20 | cattle | dry | 10 | 0.0 | 33.7 | 10.7 | 52.4 | 11-117 | 35.7 |
| | | | | | | wet | 5 | 0.0 | 17.8 | 10.9 | 61.2 | 5-71 | 17.8 |
| | | | | | sheep/goat | dry | 1 | 0.0 | 3.6 | 3.5 | 97.5 | 0.4-26 | 3.6 |
| | | | | | | wet | 2 | 0.0 | 7.1 | 4.2 | 59.2 | 2-77 | 7.2 |
| **E3c** | Halfnorm.-cosine | 31.2 | 0.22 | 104 | cattle | dry | 8 | 13.6 | 84.8 | 61.8 | 72.9 | 21-344 | 5.5 |
| | | | | | | wet | 4 | 11.5 | 52.1 | 33.8 | 64.9 | 14-196 | 2.8 |
| | | | | | sheep/goat | dry | 2 | 8.5 | 11.7 | 12.6 | 107 | 0.8-161 | 73 |
| | | | | | | wet | 0 | 0.0 | 0.0 | 0.0 | 0.0 | 0.0 | 0.0 |

| | | | | | | | | | | | | | |
|---|---|---|---|---|---|---|---|---|---|---|---|---|---|
| **E3d** | Uniform-cosine | 26.4 | 0.47 | 200 | cattle | dry | 3 | 16.3 | 17.5 | 12.6 | 72.2 | 3-89 | 1.1 |
| | | | | | | wet | 8 | 16.3 | 46.5 | 24.2 | 52.1 | 15-146 | 2.9 |
| | | | | | sheep/goat | dry | 0 | 0.0 | 0.0 | 0.0 | 0.0 | 0.0 | 0.0 |
| | | | | | | wet | 0 | 0 | 0.7 | 0.7 | 95.8 | 0-6 | 0.36 |
| **F3c** | Uniform-cosine | 26.4 | 0.47 | 120 | cattle | dry | 5 | 7.4 | 22.0 | 137.0 | 62.4 | 6-83 | 3.0 |
| | | | | | | wet | 4 | 8.0 | 19.1 | 11.3 | 59.2 | 5-68 | 2.4 |
| | | | | | sheep/goat | dry | 0 | 0.0 | 9.5 | 5.1 | 53.2 | 2.9-31 | 1.8 |
| | | | | | | wet | 3 | 5.3 | 0.0 | 0.0 | 0.0 | 0.0 | 0.0 |
| **F3d** | Uniform-cosine | 22.0 | 0.90 | 200 | cattle | dry | 3 | 12.7 | 13.6 | 14.9 | 110.2 | 2-112 | 1.1 |
| | | | | | | wet | 6 | 15.7 | 33.6 | 22.1 | 65.7 | 8-138 | 2.1 |
| | | | | | sheep/goat | dry | 0 | 0.0 | 0.0 | 0.0 | 0.0 | 0.0 | 0.0 |
| | | | | | | wet | 1 | 0 | 2.1 | 2 | 95.8 | 0.3-17 | 0.36 |
| **G3c** | Uniform-simple | 39.5 | 0.47 | 99 | cattle | dry | 6 | 17.2 | 150 | 99.4 | 66.3 | 39-572 | 4.3 |
| | | | | | | wet | 7 | 16.4 | 40.0 | 24.9 | 62.3 | 10-156 | 5.1 |
| | | | | | sheep/goat | dry | 2 | 16 | 23.1 | 21.1 | 91.4 | 3-178 | 1.4 |
| | | | | | | wet | 3 | 19.3 | 142.7 | 201.1 | 140.9 | 0-178 | 2.2 |
| **G3d** | Uniform-cosine | 27.6 | 1.00 | 101 | cattle | dry | 7 | 15.6 | 167.1 | 106.6 | 63.8 | 45-627 | 4.9 |
| | | | | | | wet | 6 | 12.0 | 23.6 | 16.2 | 56.8 | 8-165 | 4.2 |
| | | | | | sheep/goat | dry | 0 | 0.0 | 0.0 | 0.0 | 0.0 | 0.0 | 0.0 |
| | | | | | | wet | 2 | 16.0 | 22.5 | 13.0 | 57.6 | 6-86 | 1.4 |
| **H3c** | Uniform-cosine | 8.8 | 0.78 | 80 | cattle | dry | 1 | 0.0 | 8.0 | 9.0 | 112.3 | 0.81-82 | 0.9 |
| | | | | | | wet | 2 | 5.0 | 8.9 | 8.2 | 92.2 | 1-78 | 1.8 |
| | | | | | sheep/goat | dry | 1 | 0.0 | 7.1 | 8.0 | 112.2 | 0.7-73 | 1.8 |
| | | | | | | wet | 0 | 0.0 | 0.0 | 0.0 | 0.0 | 0.0 | 0.0 |
| **B5c** | Halfnorm.-cosine | 79.3 | 0.19 | 174 | cattle | dry | 15 | 17.1 | 112.0 | 65.2 | 58.1 | 30-415 | 6.2 |
| | | | | | | wet | 21 | 12.3 | 101.5 | 59.2 | 58.8 | 30-348 | 8.6 |
| | | | | | sheep/goat | dry | 2 | 19.8 | 43.4 | 57.8 | 133.2 | 24-788 | 1.6 |
| | | | | | | wet | 4 | 16.0 | 35.9 | 31.0 | 86.6 | 5.4-238 | 1.6 |
| **B5d** | Uniform-cosine | 36.0 | 0.84 | 100 | cattle | dry | 9 | 10.6 | 98.1 | 58.8 | 59.9 | 27-354 | 6.4 |
| | | | | | | wet | 9 | 6.9 | 32.6 | 19.5 | 59.7 | 10-111 | 6.4 |
| | | | | | sheep/goat | dry | 2 | 0.0 | 1.4 | 1.4 | 96.3 | 0-11 | 0.7 |
| | | | | | | wet | 1 | 0.0 | 4.3 | 3.0 | 70.6 | 1-22 | 1.4 |
| **C5c** | Halfnorm.-cosine | 44.9 | 0.33 | 101 | cattle | dry | 12 | 24.3 | 241.2 | 176.2 | 73.0 | 55-508 | 8.5 |
| | | | | | | wet | 16 | 10.4 | 105.2 | 60.5 | 57.5 | 31-352 | 11.4 |
| | | | | | sheep/goat | dry | 2 | 0.0 | 5.7 | 5.5 | 96.7 | 0.7-44 | 1.4 |
| | | | | | | wet | 8 | 2.9 | 16.9 | 10.9 | 64.7 | 4-69 | 5.7 |
| **C5d** | Uniform-cosine | 36.9 | 0.75 | 125 | cattle | dry | 7 | 6.9 | 35.2 | 28.9 | 82.3 | 7-174 | 4 |
| | | | | | | wet | 11 | 6.6 | 66.7 | 34.7 | 52.0 | 21-212 | 6.3 |
| | | | | | sheep/goat | dry | 3 | 4.0 | 11.6 | 12.3 | 106.2 | 0-343 | 1.7 |
| | | | | | | wet | 1 | 0.0 | 0.6 | 0.5 | 96.2 | 0-4.5 | 0.6 |
| **D5c** | Halfnorm.-cosine | 38.7 | 0.36 | 103 | cattle | dry | 15 | 8.5 | 104.8 | 57.7 | 55.1 | 30-363 | 10.3 |
| | | | | | | wet | 13 | 8.2 | 88.8 | 58.0 | 65.3 | 23-345 | 8.9 |
| | | | | | sheep/goat | dry | 1 | 0.0 | 3.5 | 3.9 | 113.2 | 0-34 | 0.7 |
| | | | | | | wet | 2 | 0.0 | 1.4 | 0.8 | 58.2 | 0-5 | 1.4 |
| **D5d** | Halfnorm.-cosine | 21.9 | 0.68 | 139 | cattle | dry | 7 | 12.3 | 70.9 | 61.5 | 86.7 | 13-371 | 3.6 |
| | | | | | | wet | 5 | 14.2 | 28.6 | 25.5 | 89.1 | 5-162 | 2.6 |
| | | | | | sheep/goat | dry | 0 | 0.0 | 0.0 | 0.0 | 0.0 | 0.0 | 0.0 |
| | | | | | | wet | 0 | 0.0 | 0.0 | 0.0 | 0.0 | 0.0 | 0.0 |
| **E5c** | Uniform-simple | 33.9 | 0.95 | 110 | cattle | dry | 8 | 12.9 | 117.8 | 104.1 | 88.4 | 22-632 | 5.2 |
| | | | | | | wet | 6 | 27.5 | 296.5 | 246.3 | 119.3 | 20-2116 | 3.9 |
| | | | | | sheep/goat | dry | 1 | 0.0 | 1.9 | 1.8 | 96.5 | 0-15 | 0.6 |
| | | | | | | wet | 4 | 15.3 | 123.2 | 148.6 | 120.6 | 9-1674 | 2.6 |
| **E5d** | Uniform- | 43.7 | 0.45 | 122 | cattle | dry | 10 | 14.2 | 113.4 | 62.5 | 55.1 | 34-384 | 5.8 |

| | | | | | | | | | | | | | |
|---|---|---|---|---|---|---|---|---|---|---|---|---|---|
| | simple | | | | | | | | | | | | |
| | | | | | | wet | 8 | 12.0 | 57.1 | 34.5 | 60.4 | 16-201 | 4.7 |
| | | | | | sheep/goat | dry | 1 | 0.0 | 1.7 | 2.0 | 113.0 | 0-18 | 0.6 |
| | | | | | | wet | 3 | 10.0 | 3.2 | 3.1 | 97.1 | 0-26 | 1.8 |
| **F5c** | Uniform-simple | 37.1 | 0.54 | 126 | cattle | dry | 7 | 11.7 | 60.9 | 46.2 | 75.7 | 14-271 | 3.9 |
| | | | | | | wet | 9 | 13.6 | 77.4 | 48.4 | 62.4 | 21-282 | 5.1 |
| | | | | | sheep/goat | dry | 0 | 0.0 | 0.0 | 0.0 | 0.0 | 0.0 | 0.0 |
| | | | | | | wet | 2 | 2.5 | 2.8 | 1.7 | 67.5 | 0-1.1 | 1.1 |
| **F5d** | Uniform-cosine | 61.1 | 0.35 | 114 | cattle | dry | 10 | 11.7 | 84.3 | 49.6 | 59.8 | 24-290 | 6.3 |
| | | | | | | wet | 9 | 13.6 | 76.8 | 45.4 | 59.2 | 23-262 | 5.6 |
| | | | | | sheep/goat | dry | 5 | 7.4 | 21.6 | 18.4 | 85.1 | 4 - 118 | 3.1 |
| | | | | | | wet | 4 | 4.3 | 34.1 | 42.4 | 124.1 | 2 - 487 | 2.5 |
| **G5c** | Uniform-cosine | 48.3. | 0.42 | 150 | cattle | dry | 10 | 13.0 | 61.9 | 41.5 | 67.0 | 14-277 | 4.8 |
| | | | | | | wet | 7 | 14.1 | 47.1 | 35.6 | 75.4 | 9 - 247 | 3.3 |
| | | | | | sheep/goat | dry | 1 | 0.0 | 6.2 | 5.9 | 95.8 | 0.7-49 | 0.5 |
| | | | | | | wet | 4 | 21.8 | 41.4 | 33.6 | 81.0 | 7.5-229 | 1.9 |
| **G5d** | Uniform-cosine | 24.2 | 0.53 | 200 | cattle | dry | 3 | 19.7 | 21.1 | 15.6 | 73.8 | 4 -107 | 1.1 |
| | | | | | | wet | 5 | 26.2 | 46.8 | 26.0 | 55.6 | 14-154 | 1.7 |
| | | | | | sheep/goat | dry | 1 | 0.0 | 18.9 | 18.1 | 95.8 | 2-151 | 0.4 |
| | | | | | | wet | 2 | 9.5 | 6.8 | 5.7 | 84.5 | 1-44 | 0.7 |
| **H5c** | Uniform-cosine | 25.0 | 0.74 | 200 | cattle | dry | 4 | 13.0 | 18.6 | 10.9 | 58.8 | 5-74 | 1.4 |
| | | | | | | wet | 4 | 8.0 | 11.4 | 7.9 | 69.0 | 2-56 | 1.4 |
| | | | | | sheep/goat | dry | 0 | 0.0 | 0.0 | 0.0 | 0.0 | 0.0 | 0.0 |
| | | | | | | wet | 1 | 0.0 | 2.5 | 2.4 | 95.8 | 0-20 | 0.4 |
| **H5d** | Halfnorm.-cosine | 17.7 | 0.54 | 112 | cattle | dry | 3 | 11.3 | 39.7 | 40.6 | 102.4 | 5-305 | 1.9 |
| | | | | | | wet | 1 | 0.0 | 13.9 | 13.9 | 99.9 | 2-108 | 0.6 |
| | | | | | sheep/goat | dry | 2 | 11.0 | 13.9 | 14.4 | 103.3 | 0.3-313 | 1.3 |
| | | | | | | wet | 3 | 11.7 | 12.5 | 12.4 | 107.7 | 0-418 | 1.7 |
| **I5c** | Halfnorm.-cosine | 22.3 | 0.08 | 44 | cattle | dry | 3 | 33.3 | 246.9 | 215.9 | 87.4 | 40-1508 | 4.9 |
| | | | | | | wet | 5 | 15.2 | 147.7 | 115.0 | 77.9 | 32-286 | 8.2 |
| | | | | | sheep/goat | dry | 0 | 0.0 | 0.0 | 0.0 | 0.0 | 0.0 | 0.0 |
| | | | | | | wet | 3 | 3.7 | 26.8 | 25.8 | 96.3 | 2.4-304 | 4.9 |
| **B9a** | Uniform-cosine | 15.4 | 0.87 | 40 | cattle | dry | 1 | 0.0 | 1.8 | 1.7 | 95.8 | 0-14 | 1.8 |
| | | | | | | wet | 1 | 0.0 | 12.5 | 11.9 | 95.8 | 2-99 | 1.8 |
| | | | | | sheep/goat | dry | 2 | 5.0 | 17.9 | 10.7 | 59.9 | 5-70 | 3.6 |
| | | | | | | wet | 3 | 2.3 | 12.5 | 10.7 | 96.9 | 2-99 | 5.4 |
| **B9b** | Halfnorm.-cosine | 19.3 | 0.11 | 16 | cattle | dry | 3 | 0.0 | 13.2 | 13.0 | 99.1 | 1 - 102 | 13.2 |
| | | | | | | wet | 1 | 0.0 | 8.8 | 8.7 | 99.1 | 1-68 | 4.4 |
| | | | | | sheep/goat | dry | 5 | 2.6 | 33.2 | 25.9 | 78.0 | 7-155 | 21.9 |
| | | | | | | wet | 1 | 0.0 | 30.8 | 35.4 | 115.1 | 4-306 | 4.4 |
| **C9a** | Uniform-cosine | 11.0 | 0.82 | 16 | cattle | dry | 2 | 3.5 | 31.3 | 22.1 | 70.6 | 6-167 | 8.9 |
| | | | | | | wet | 2 | 3.5 | 31.3 | 25.6 | 82.1 | 5-187 | 8.9 |
| | | | | | sheep/goat | dry | 1 | 0.0 | 35.7 | 34.2 | 95.8 | 5-284 | 4.5 |
| | | | | | | wet | 0 | 0.0 | 0.0 | 0.0 | 0.0 | 0.0 | 0.0 |
| **C9b** | Uniform-cosine | 8.8 | 0.78 | 25 | cattle | dry | 1 | 0.0 | 8.5 | 9.6 | 112.3 | 1-87 | 2.9 |
| | | | | | | wet | 0 | 0.0 | 0.0 | 0.0 | 0.0 | 0.0 | 0.0 |
| | | | | | sheep/goat | dry | 2 | 6.0 | 34.3 | 33.4 | 97.3 | 4-270 | 5.7 |
| | | | | | | wet | 1 | 0.0 | 14.3 | 16.0 | 112.3 | 1-145 | 2.9 |
| **D9a** | Halfnorm.-cosine | | | | cattle | dry | 0 | 0.0 | 0.0 | 0.0 | 0.0 | 0.0 | 0.0 |
| | | | | | | wet | 0 | 0.0 | 0.0 | 0.0 | 0.0 | 0.0 | 0.0 |
| | | | | | sheep/goat | dry | 0 | 0.0 | 0.0 | 0.0 | 0.0 | 0.0 | 0.0 |
| | | | | | | wet | 0 | 0.0 | 0.0 | 0.0 | 0.0 | 0.0 | 0.0 |
| **D9b** | Uniform-cosine | 26.4 | 0.47 | 180 | cattle | dry | 3 | 24.3 | 29.0 | 24.3 | 84.0 | 5-163 | 1.2 |

| | | | | | | | | | | | | | |
|---|---|---|---|---|---|---|---|---|---|---|---|---|---|
| | | | | | | wet | 6 | 9.0 | 21.4 | 13.4 | 62.5 | 6-78 | 2.4 |
| | | | | | sheep/goat | dry | 0 | 0.0 | 0.0 | 0.0 | 0.0 | 0.0 | 0.0 |
| | | | | | | wet | 3 | 6.3 | 7.5 | 5.19 | 77.7 | 1.5-39 | 1.2 |
| **E9a** | Uniform-cosine | 35.2 | 0.65 | 200 | cattle | dry | 7 | 17.9 | 44.6 | 31.9 | 71.5 | 8-205 | 2.5 |
| | | | | | | wet | 6 | 17.9 | 38.2 | 25.5 | 66.6 | 9-162 | 2.1 |
| | | | | | sheep/goat | dry | 2 | 0.0 | 5.7 | 5.5 | 95.8 | 0.7-46 | 0.7 |
| | | | | | | wet | 1 | 0.0 | 3.2 | 3.1 | 95.8 | 0-26 | 0.4 |
| **E9b** | Halfnorm.-cosine | 34.1 | 0.41 | 124 | cattle | dry | 4 | 9.5 | 23.5 | 24.5 | 104.7 | 3-189 | 20.0 |
| | | | | | | wet | 9 | 6.0 | 15.3 | 9.7 | 63.6 | 4-55 | 4.5 |
| | | | | | sheep/goat | dry | 1 | 0.0 | 1.5 | 1.7 | 114.9 | 0-15 | 0.5 |
| | | | | | | wet | 2 | 3.0 | 3.0 | 2.8 | 93.0 | 0-32 | 1.0 |
| **F9a** | Uniform-cosine | 37.4 | 0.39 | 200 | cattle | dry | 4 | 11.0 | 15.7 | 11.9 | 76.0 | 3-86 | 1.4 |
| | | | | | | wet | 5 | 14.0 | 25.0 | 15.1 | 60.5 | 6-100 | 1.8 |
| | | | | | sheep/goat | dry | 4 | 5.0 | 7.1 | 5.3 | 74.1 | 1.5-35 | 1.4 |
| | | | | | | wet | 4 | 5.0 | 7.1 | 4.8 | 66.7 | 2-29 | 1.4 |
| **F9b** | Halfnorm.-simple | 29.3 | 0.01 | 60 | cattle | dry | 6 | 10.0 | 66.3 | 43.1 | 65.0 | 17-258 | 7.1 |
| | | | | | | wet | 10 | 7.9 | 70.7 | 32.8 | 56.2 | 20-240 | 11.8 |
| | | | | | sheep/goat | dry | 2 | 3.0 | 7.1 | 4.8 | 68.1 | 2-31 | 2.4 |
| | | | | | | wet | 1 | 0.0 | 7.1 | 6.9 | 97.6 | 1-56 | 1.2 |
| **G9a** | Halfnorm.-cosine | 45.8 | 0.78 | 67 | cattle | dry | 7 | 10.9 | 120.6 | 115.7 | 96.0 | 20-717 | 7.4 |
| | | | | | | wet | 8 | 6.3 | 62.9 | 46.0 | 73.2 | 15-271 | 8.5 |
| | | | | | sheep/goat | dry | 2 | 0.0 | 4.3 | 4.2 | 97.6 | 0.5-33 | 2.1 |
| | | | | | | wet | 6 | 8.2 | 27.4 | 25.7 | 93.8 | 45-167 | 6.4 |
| **G9b** | Halfnorm.-simple | 22.5 | 0.20 | 31 | cattle | dry | 6 | 12.0 | 134.5 | 150.8 | 112.2 | 17-1064 | 13.9 |
| | | | | | | wet | 3 | 6.7 | 40.3 | 59.9 | 148.5 | 1-1409 | 6.9 |
| | | | | | sheep/goat | dry | 3 | 2.7 | 159.1 | 2856 | 179.5 | 0-9800 | 6.9 |
| | | | | | | wet | 3 | 3.0 | 17.9 | 13.2 | 73.5 | 4-85 | 6.9 |
| **H9a** | Uniform-cosine | 54.9 | 0.23 | 140 | cattle | dry | 11 | 12.5 | 69.9 | 42.1 | 60.3 | 18-269 | 5.6 |
| | | | | | | wet | 9 | 13.2 | 60.7 | 37.2 | 61.2 | 17-235 | 4.6 |
| | | | | | sheep/goat | dry | 2 | 8.5 | 8.7 | 5.5 | 63.6 | 2-36 | 1.0 |
| | | | | | | wet | 3 | 7.7 | 11.7 | 7.6 | 64.9 | 3-51 | 1.5 |
| **H9b** | Uniform-simple | 26.6 | 0.87 | 68 | cattle | dry | 4 | 27.5 | 965.4 | 1221.1 | 198.9 | 12-8011 | 4.2 |
| | | | | | | wet | 7 | 29.5 | 305.7 | 327.9 | 107.3 | 42-2224 | 7.3 |
| | | | | | sheep/goat | dry | 3 | 8.0 | 74.0 | 104.9 | 141.7 | 0-18310 | 3.1 |
| | | | | | | wet | 0 | 0.0 | 0.0 | 0.0 | 0.0 | 0.0 | 0.0 |
| **I9a** | Uniform-cosine | 24.2 | 0.91 | 130 | cattle | dry | 5 | 28.2 | 77.4 | 54.4 | 70.2 | 18-326 | 2.7 |
| | | | | | | wet | 3 | 47.3 | 78.0 | 59.9 | 76.8 | 15-392 | 1.6 |
| | | | | | sheep/goat | dry | 2 | 12.0 | 13.2 | 12.8 | 97.3 | 1-103 | 1.1 |
| | | | | | | wet | 1 | 0.0 | 3.3 | 3.1 | 95.8 | 0-26 | 0.5 |

**Questionnaire 1**

Semi-structured questionnaire with questions asked to the respondents regarding the persistence and abundance of wildlife in the Mutara rangelands. Note that only the answers to questions 4 were analysed in this study

**Questionnaire**

*(The interviewees will be asked the following questions strictly in the given order)*

**1.** At first we will request the interviewees to provide information on **age, education level, gender, occupation** (what do you do on this land)**, origin *(local or stranger).***
**2.** What wildlife species do you see (hear)?

**Silhouettes will be presented to the interviewee**

**3.** When did you last encounter that species on your land?
**4.** How often do you see that species?
**5.** Does this wild animal cause any damage?
**6.** What damage?
**7.** If crop is raided: What crop/livestock is raided?
**8.** How do you estimate the damage in % of expected yield?
**9.** Can you tolerate the species to be with your livestock/on your land?
**10.** Do you try to repel the wild animal?
**11.** In what habitat do you encounter this wild animal (garden, cattle ranch, riverine forest, bush and tickets)
